# Does the Use of Nitroglycerin Patch Improve Local Anaesthetic Effects in Bier's Block? A Double-Blind Placebo Controlled Study

**DOI:** 10.1155/2018/9674731

**Published:** 2018-03-07

**Authors:** Ahmed Said Elgebaly

**Affiliations:** Department of Anesthesia and Postsurgical Intensive Care, Faculty of Medicine, Tanta University, Tanta, Egypt

## Abstract

**Aim:**

The aim of the study was to assess the nitroglycerin patch as a new additive to Bier's block and its impact on the effects and dose of lidocaine.

**Methods:**

Forty patients of each sex belonging to ASA I or II underwent elective tendon repair surgeries of the forearm and hand. The patients were divided into two equal groups as follows: Group C received only lidocaine (1.5 mg/kg, 0.25%) and Group N received lidocaine (1.5 mg/kg, 0.25%) + 5 mg transcutaneous nitroglycerin patch. Onset and recovery times for sensory and motor block, visual analogue scale (VAS) scores for bandage pain, postoperative VAS score, analgesic requirements, patients' satisfaction, and surgeons' opinion were recorded.

**Results:**

Sensory block onset time was shorter in Group N (3.80 ± 1.0) than that in Group C (5.72 ± 1.46), and motor block onset time was shorter in Group N (10.72 ± 1.93) than that in Group C (13.56 ± 1.26). Sensory block recovery time was prolonged in Group N (10.56 ± 1.12) than Group C (6.88 ± 1.45), recovery time of motor block was prolonged in Group N (13.04 ± 1.57) than Group C (11.96 ± 1.72). Bandage pain had lower VAS scores in Group N. Postoperative VAS scores showed significant differences between both groups at the following points of measurement: 30 minutes, 1 hour, and 4 hours after bandage deflation. Postoperative analgesic effect was the longest in Group N (187.20 ± 60.79 min) than Group C (51.60 ± 25.28 min). Patients' satisfaction and surgeons' opinion were better in Group N than Group C.

**Conclusion:**

Supplementation of Bier's block with transcutaneous nitroglycerin patch reduces the lidocaine dose, the sensory and motor block onset times, VAS scores, and analgesic consumption intra- and postoperatively. Length of the block recovery times for the sensory and motor effects, duration of postoperative analgesic effect, and the first time to analgesic requirement improved the quality of Bier's block with better patients' satisfaction and surgeons' opinion and had no adverse effects.

## 1. Introduction

Bier's block is a technique that is mostly used for providing anaesthesia and analgesia during the operation. Using additives with lidocaine may provide satisfactory anaesthesia and prolong the duration of postoperative analgesia [1].

Intravenous regional anaesthesia (IVRA) which was firstly described by August Bier in 1902 [2] is used successfully as a sole technique for upper limb surgeries [3]. It is a reliable, simple, cost-effective, and widely accepted technique of regional anaesthesia [1].

Disadvantages of Bier's block include the potential for local anaesthetic toxicity, emergence of pain following removal of the bandage (within three to five minutes), and lack of postoperative analgesia [4, 5]. Several adjuncts have been used including narcotics, nonsteroidal anti-inflammatory drugs (NSAIDs), muscle relaxants, *α*_2_ agonists, and neostigmine [6].

Nitroglycerin (NTG) and nitric oxide generator contained in transcutaneous nitroglycerin help the distribution and absorption of local anaesthetic agents to neuron and trunks by vasodilatation [7, 8]. The aim of the present study was to evaluate whether the supplementation of 5 mg nitroglycerin patch to low-dose lidocaine will improve the overall Bier's block effect either intra- or postoperatively as primary outcomes and analgesic requirements, patients' satisfaction, and surgeons' opinion as secondary outcomes.

## 2. Materials and Methods

After obtaining the approval from the native Ethics and Research Committee, written informed consent from all the study participants was obtained. A double-blind prospective randomized study enrolled 40 ASA physical status I or II patients of each sex, aged ≥18 years and undergoing elective tendon repair surgeries of the forearm. Patients who refused; patients with Reynauld's disease, sickle-cell disease, crush injuries, and swelling or skin infection at the site of injection; and patients with a history of allergy to nitroglycerin or lidocaine and surgeries >60 minutes or <30 minutes were excluded from the study.

After history taking, complete clinical examination and laboratory investigations, monitoring of the patient with noninvasive blood pressure (NIBP), ECG, and peripheral oxygen saturation was performed. A peripheral venous line with 20-Gauge cannula was inserted in the contralateral hand for crystalloid infusion.

Randomization by use of sealed envelopes and allocation of patients to two equal groups were done as follows:

 
*Group C (control group)*: 40 cc of 1.5 mg/kg of 0.25% lidocaine diluted in normal saline (0.9% NaCl) was used for administering Bier's block. Empty nitroglycerin patch was applied on the ventral aspect of the proximal forearm of the operative arm 2 hours preoperatively.
 
*Group N (lidocaine and transdermal nitroglycerin patch group)*: 40 cc of 1.5 mg/kg of 0.25% lidocaine diluted in normal saline (0.9% NaCl) with application of 5 mg of transcutaneous nitroglycerin patch was used for administering Bier's block. Nitroglycerin patch was applied on the ventral aspect of the proximal forearm of the operative arm 2 hours preoperatively. Another peripheral venous line with 22-Gauge cannula was inserted in the dorsum of the operative hand (as distal as possible) for injection of the study drugs.

A double pneumatic bandage was placed on the upper arm with generous layers of cotton padding on the operative side ensuring that no wrinkles are formed and the bandage edges do not touch the skin, and then it was exsanguinated by 2-minute elevation and wrapping with an Esmarch bandage.

Before lidocaine was injected, circulatory isolation of the arm was verified by inspection, absence of radial pulse, and loss of pulse oximetry tracing in the ipsilateral index finger, and then the lidocaine solution was injected by inflation to a minimum of 250 mmHg for bandage lying at the proximal site.

Block onset was estimated after ten minutes of local anaesthetic administration. Distal bandage was inflated up to 250 mmHg after fifteen minutes, and the proximal bandage was deflated after taking out transdermal nitroglycerin patch. If no block occurs up to 15 minutes, the patient was excluded and replaced with another matched one. Deflation of cuff was not done until thirty minutes after lidocaine injection passed because systemic toxicity of lidocaine may occur and was not inflated for more than one and a half hours.

Assessment of demographic data, duration of surgery, sensory and motor block onset and recovery times, onset time of bandage pain and number of patients complaining from it, and intraoperative and 24-hour postoperative analgesic requirements was performed. Postoperative VAS score and first time of analgesic requirement were recorded.

Patients' satisfaction was obtained by asking the patient to rate the operative conditions according to the following numerical scale: 0 = unsuccessful, 1 = poor, 2 = moderate (pain required supplemental analgesia), 3 = good (minor pain with no need of supplemental analgesia), and 4 = excellent (no pain). Also surgeons' opinion was also obtained. As surgeons were blind to randomization of the patients, they rate the conditions of the operation with regard to movement of limb or bleeding, and the scores were as follows: 4 = excellent, 3 = good, 2 = acceptable, 1 = poor, and 0 = unsuccessful. Complications such as CNS (circum oral numbness, headache, convulsions, or coma) or CVS (bradycardia, arrhythmia, or hypotension) were recorded.

### 2.1. Statistical Analysis

IBM SPSS software package version 20.0 was used for analysis of the collected data. Mean ± standard deviation (SD) was used to express the quantitative data. Frequency and percentage were used to express the qualitative data. When comparing between two means, the independent-samples *t*-test of significance was used. In order to compare proportions between two qualitative parameters, chi-square (*χ*^2^) test of significance was used.

## 3. Results

Age, sex, weight, ASA physical status, duration, and type of surgery showed insignificant difference with regard to comparable demographic data (Table 1).

Sensory block onset time was shorter in Group N (3.80 ± 1.0) than Group C (5.72 ± 1.46), and motor block onset time was shorter in Group N (10.72 ± 1.93) than Group C (13.56 ± 1.26). Sensory block recovery time was prolonged in Group N (10.56 ± 1.12) than Group C (6.88 ± 1.45), and recovery of motor block time in Group N was prolonged (13.04 ± 1.57) than Group C (11.96 ± 1.72) (Table 2).

VAS scores for bandage pain showed significant differences between the two groups at the following points of measurement: 10, 15, 20, and 30 minutes after tourniquet inflation. Also, there was a significant difference in the number of patients complained of bandage pain between the two groups: in Group C, 52% of patients complained of bandage pain, whereas in Group N, only 24% of patients complained of bandage pain. The onset time of bandage pain was longer in Group N (29.17 ± 2.04 min) than Group C (21.54 ± 3.15 min) (Table 3).

**Figure 1:**
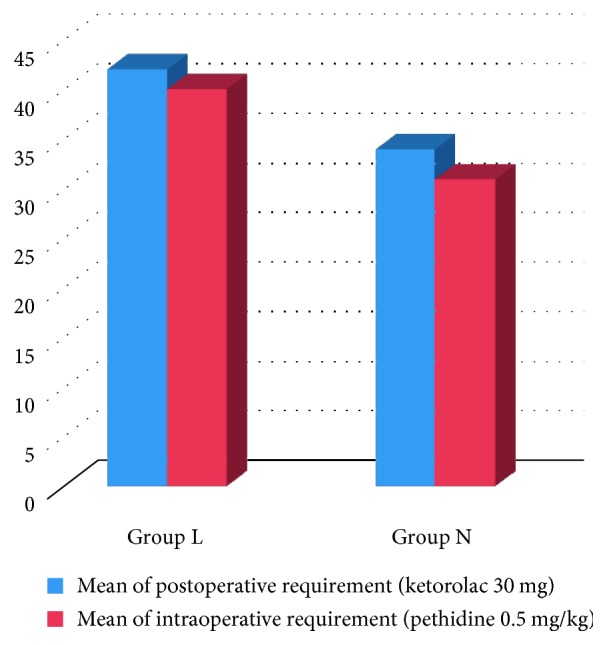
Intra- and postoperative analgesic requirements for both groups.

Postoperative VAS scores showed significant differences between the two groups at the following points of measurement: 30 minutes, 1 hour, and 4 hours after bandage deflation, with lower VAS scores in Group N (1.36 ± 0.81, 1.92 ± 0.40) (Table 4). Postoperative analgesic effect was longer in Group N (187.20 ± 60.79 min) than Group C (51.60 ± 25.28 min). Postoperative analgesic requirement was the lowest in Group N (33.60 ± 9.95 mg) (Table 5) (Figure 1).

In the present study, the patients' satisfaction about the operation and the surgeons' opinion about the operative conditions were better in Group N than Group C.

No adverse effects or complications were seen in this study. No evidence of central nervous system complications (such as circum oral numbness, headache, convulsions, or coma) or cardiac complications (such as arrhythmias, hypotension, or bradycardia) was seen after lidocaine administration, before and during the surgery, and after release of the bandage.

## 4. Discussion

Our study results showed that the addition of 5 mg transcutaneous nitroglycerin patch as adjuvant to Bier's block reduced the lidocaine dose to 1.5 mg/kg of 0.25% lidocaine; reduced the onset times of sensory and motor block, VAS scores, and analgesic consumption; prolonged the sensory and motor block recovery times and the duration of postoperative analgesic effect of Bier's block; delayed the first time to analgesic requirement; and improved the quality of Bier's block with no adverse effects.

The mechanism of action of nitroglycerin seems to be augmented by the direct potent vasodilatory effect that allows distribution of lidocaine to nerves that is mainly dose-dependent [9]. Several studies explained the pain-relieving mechanism of nitroglycerin [10, 11]. Nitroglycerin produces its analgesic effect by metabolization in the cell to nitric oxide, which causes an increase in the intracellular concentration of cyclic guanosine monophosphate, which produces pain modulation in the nervous systems (central and peripheral). Topical application of nitroglycerin generators also induces analgesia and anti-inflammatory effects by blocking the neurogenic component of inflammatory edema and hyperalgesia [12].

The first study on adding nitroglycerin to lidocaine to Bier's block for hand and forearm surgery was performed by Sen et al. [13] who studied the effect of adding nitroglycerin to lidocaine to Bier's block on 30 patients undergoing hand surgery in two groups: Group C, *n*=15, as the control group which received 3 mg/kg of lidocaine diluted with saline, a total dose of 40 mL, and Group NTG, *n*=15, as the nitroglycerin group in which patients received an additional 200 *μ*g of nitroglycerin.

In agreement with our results, Sen et al. [13] proved that there was hemodynamic stability in the perioperative period, shortened onset time of sensory and motor blocks, higher VAS scores of bandage pain with prolonged sensory block recovery time, and improved quality of anaesthesia in the nitroglycerin group, whereas VAS scores were lower in the nitroglycerin group in the postoperative period. First analgesic requirement time was longer in the nitroglycerin group than in Group C. In the nitroglycerin group, requirements for analgesia postoperative were significantly smaller.

Similar results were obtained by various other authors. Cakmak et al. [5] studied 60 patients undergoing elective hand, wrist, and forearm surgery procedures distributed into three groups: Group L: lidocaine group whichreceived 3 mg/kg of 2% lidocaine to a total volume of 40 mL and was diluted with saline; Group LL: lidocaine and lornoxicam group which received 3 mg/kg of 2% lidocaine to a total volume of 40 mL and was diluted with saline and also 8 mg of lornoxicam was added to the solution; and Group LL-N: lidocaine with lornoxicam and transdermal nitroglycerin group in which nitroglycerin patch 5 mg was applied to the surgical site and received 3 mg/kg of 2% lidocaine diluted with saline to a total volume of 40 mL and also 8 mg of lornoxicam was added to the solution, and they found shortened sensory and motor block onset times and prolonged sensory and motor block recovery times in the group which contained the nitroglycerin patch and lidocaine with lornoxicam than the lidocaine-alone group.

Previous researches did not study the patients' satisfaction, surgeons' opinion, and adverse effects or complications. In the present study, we focused on these parameters and found that the patients' satisfaction about the operation and surgeons' opinion of the operative conditions were better in Group N than Group C with no adverse effects or complications seen in this study.

Contrary to Cakmak et al. [5], although we used the transcutaneous nitroglycerin as they did, in our study we got the same results with low lidocaine concentration (40 cc of 1.5 mg/kg of 0.25% lidocaine diluted in normal saline (0.9% NaCl) was used for administering Bier's block). Low lidocaine concentration was the primary and effective cause for decreased resulted adverse effects and complications intra- and postoperatively and increased patients' satisfaction and surgeons' opinion in our study.

Also, Asadi and Mehri [14] studied the analgesic effect of nitroglycerin when added to lidocaine for Bier's block on 40 patients scheduled for elective forearm and hand surgery and agreed our results by reporting shorter sensory block onset time and delay in sensory block recovery time after bandage release in the nitroglycerin group with shorter motor block onset time and delay in recovery time after bandage release in the first group; the frequency of opioid injections was significantly lower in those who administered lidocaine and nitroglycerin.

Elmetwaly et al. [6] studied the effect of adding ketamine or nitroglycerin to lidocaine for Bier's block on seventy-five patients undergoing hand surgery and found that, in the control group, the starting four hours postoperatively, the VAS scores were higher compared with the other study groups. Also, Cakmak et al. [5] noted and agreed these results by approving that, in Group C, the starting four hours postoperatively, the VAS scores were higher in comparison to other groups.

## 5. Study Limitations and Recommendations

The major limitation of the present study was the current small sample size since we were able to enroll only 40 patients. More studies with high and sufficient sample sizes are required to confirm these results. We recommend further larger studies to determine the effect of different doses of lidocaine and nitroglycerin and other additives that can affect Bier's block conditions.

## 6. Conclusion

From the results of the present study, we conclude that, the addition of 5 mg of transcutaneous nitroglycerin patch as supplementation to 1.5 mg/kg of 0.25% lidocaine for Bier's block reduced the dose of lidocaine, sensory and motor block onset times, VAS scores, and analgesic consumption. Length of the sensory and motor block recovery times, the duration of postoperative analgesic effect, and the first time to analgesic requirement improved the quality of Bier's block with better patients' satisfaction and surgeons' opinion and had no side effects.

## Figures and Tables

**Table 1:** Demographic data.

	Group C (*n*=20)	Group N (*n*=20)	*P* value
Age (years)	31.28 ± 9.91	28.36 ± 9.31	0.637
Sex (%)			0.951
M	60	64	
F	40	36	
Weight (kg)	72.24 ± 12.53	68.40 ± 9.97	0.202
ASA (%)			1.000
I	84	88	
II	16	12	
Duration of surgery (min)	44.92 ± 8.30	45.48 ± 8.16	0.980

Data are represented as mean ± SD; yr: year; kg: kilogram; min: minute; M: male; F: female.

**Table 2:** Sensory and motor block onset and recovery times.

	Group C (*n*=20)	Group N (*n*=20)	*P* value
Sensory block onset time (min)	5.72 ± 1.46	3.80 ± 1.0	<0.001^∗^
Motor block onset time (min)	13.56 ± 1.26	10.72 ± 1.93	<0.001^∗^
Sensory block recovery time (min)	6.88 ± 1.45	10.56 ± 1.12	<0.001^∗^
Motor block recovery time (min)	11.96 ± 1.72	13.04 ± 1.57	<0.001^∗^

Data are represented as mean ± SD; ^∗^statistically significant at *P* < 0.05.

**Table 3:** VAS scores for tourniquet pain.

	Group C (*n*=20)	Group N (*n*=20)	*P* value
5 min	0.68 ± 0.69	0.52 ± 0.59	0.065
10 min	1.44 ± 0.77	0.68 ± 0.63	<0.001^∗^
15 min	2.24 ± 0.52	1.04 ± 0.35	<0.001^∗^
20 min	3.28 ± 0.79	1.44 ± 0.58	<0.001^∗^
25 min	2.68 ± 0.69	2.12 ± 0.78	<0.001^∗^
30 min	2.44 ± 0.58	2.96 ± 0.61	0.001^∗^
End of surgery	2.24 ± 0.44	2.52 ± 0.51	0.099

Data are represented as mean ± SD; ^∗^statistically significant at *P* < 0.05; min: minutes.

**Table 4:** Postoperative VAS score.

	Group C (*n*=20)	Group N (*n*=20)	*P* value
30 min	2.96 ± 1.06	1.36 ± 0.81	<0.001^∗^
1 hr	2.96 ± 1.02	1.92 ± 0.40	<0.001^∗^
2 hr	2.08 ± 0.64	2.56 ± 1.42	0.243
4 hr	2.16 ± 0.47	3.12 ± 1.05	<0.001^∗^
6 hr	2.0 ± 0.29	2.0 ± 0.50	0.918
8 hr	2.08 ± 0.49	2.20 ± 0.41	0.698
12 hr	1.68 ± 1.46	1.88 ± 0.60	0.439
16 hr	1.44 ± 1.29	1.76 ± 0.83	0.189
20 hr	0.76 ± 1.09	0.76 ± 1.16	0.593
24 hr	0.44 ± 0.77	0.44 ± 0.71	0.254

Data are represented as mean ± SD; ^∗^statistically significant at *P* < 0.05; hr: hours.

**Table 5:** Analgesic requirements.

*P* value	Group C (*n*=20)	Group N (*n*=20)	*P* value
Intraoperative (pethidine 0.5 mg/kg)	39.23 ± 6.07	31.67 ± 5.16	0.002^∗^
Postoperative (ketorolac 30 mg)	40.80 ± 14.70	33.60 ± 9.95	0.013^∗^

Data are represented as mean ± SD; ^∗^statistically significant at *P* < 0.05; mg: milligram; kg: kilogram.
